# The Accidental Dislodgement of Renal Calculi

**Published:** 1901-12-21

**Authors:** 


### THE ACCIDENTAL DISLODGEMENT OF RENAL CALCULI.

In the Bradshaw Lecture delivered before the Royal College of Surgeons Mr. T. R. Jessop relates many very interesting cases bearing upon the surgery of the kidney. Some of these show in a curious way how stone in the kidney may sometimes be dislodged by pressure or manipulation applied externally. One was the case of a woman who had suffered so much that she had required the daily administration of morphia for a period of from three to four months. Eventually, during the night following an examination which included a free manipulation of the loin, she passed a small lithic acid calculus by the urethra, to her complete and permanent relief. Another case was that of a man who had been injured in a railway accident. Among other details of the accident it was stated that he was thrown upon the floor of the compartment, where he received several blows upon the back from other passengers falling in a confused heap upon him. After having been conveyed home, his sufferings were so severe that, pending the arrival of a medical man, his wife had him placed in a hot bath. Whilst in the bath, micturition became imperative and frequent, the urine being decidedly blood-stained, and at length he emitted a lithic calculus of the size and shape of a barleycorn. Another case was that of a young man suffering from well-marked renal colic. His kidney was examined by careful manipulation, and kidney puncture. The search was abandoned, and the wound was closed. Early the next day, however, he was found to be suffering from retention of urine due to the impaction of a calculus in the membranous urethra plainly felt in the perineum. This was removed by a median incision, and the retention was speedily relieved.

